# Early detection of plasma d-lactate: Toward a new highly-specific biomarker of bacteraemia?

**DOI:** 10.1016/j.heliyon.2023.e16466

**Published:** 2023-05-21

**Authors:** Charles R. Lefèvre, Adrien Turban, David Luque Paz, Malo Penven, Céline René, Bénédicte Langlois, Maxime Pawlowski, Nicolas Collet, Caroline Piau, Vincent Cattoir, Claude Bendavid

**Affiliations:** a Biochemistry Laboratory, Pontchaillou Hospital, Rennes University Hospital, Rennes, France; b Bacteriology Laboratory, Pontchaillou Hospital, Rennes University Hospital, Rennes, France; c Infectious Diseases and Intensive Care Unit, Pontchaillou Hospital, Rennes University Hospital, Rennes, France; d Microbiology Department, University Hospital of Caen, Caen, France

**Keywords:** D-lactate, Bacteraemia, Specific, Biomarker, Sepsis, Infection

## Abstract

**Background:**

Bloodstream infections are a leading cause of mortality. Their detection relies on blood cultures (BCs) but time to positivity is often between tens of hours and days. d-lactate is a metabolite widely produced by bacteria but very few in human. We aimed to evaluate d-lactate, d-lactate/l-lactate ratio and d-lactate/total lactate ratio in plasma as potential early biomarkers of bacteraemia on a strictly biological standpoint.

**Methods:**

A total of 228 plasma specimens were collected from patients who had confirmed bacteraemia (*n* = 131) and healthy outpatients (*n* = 97). Specific l-lactate and d-lactate analyses were performed using enzymatic assays and analytical performances of d-lactate, d-lactate/total lactate and d-lactate/l-lactate ratios for the diagnosis of bacteraemia were assessed.

**Results:**

A preliminary *in vitro* study confirmed that all strains of *Escherichia coli*, *Klebsiella pneumoniae* and *Staphylococcus aureus* were able to produce d-lactate at significant levels. In patients, plasma d-lactate level was the most specific biomarker predicting a bacteraemia profile with a specificity and predictive positive value of 100% using a cut-off of 131 μmol.L^−1^. However, sensitivity and negative predictive value were rather low, estimated at 31% and 52%, respectively. d-lactate displayed an Area Under Receiver Operating Characteristic (AUROC) curve of 0.696 with a P value < 0.0001. There was no difference of d-lactate levels between BCs bottles positive for Gram-positive or Gram-negative bacteria (p = 0.55).

**Conclusion:**

d-lactate shows promise as a specific early biomarker of bacterial metabolism. The development of rapid automated assays could raise clinical applications for infectious diseases diagnosis including early bacteraemia prediction.

## 1 Introduction

Lactic acid (*i.e*. 2-hydroxypropanoic acid, C_3_H_6_O_3_) is a 90-Da metabolite and the second most abundant circulating carbon carrier in mammals after glucose, from 1000 to 2000 μmol.L^−1^ in plasma [1]. This organic acid (pKa = 3.86 at 20 °C) is metabolized from pyruvic acid by the lactate dehydrogenase (LDH, enzyme commission number: EC 1.1.1.27), while also producing nicotinamide adenine dinucleotide (NAD). The β carbon atom of lactic acid being asymmetric, there are two enantiomers: (R)-lactic acid (or l(+)-lactate), the most abundant, and (S)-lactic acid (or d(−)-lactate) that is marginal in vertebrates ([l-lactate]:[d-lactate] ratio = 100:1) [2,3]. For both enantiomers, specific LDHs have been described [4,5]. l-lactate dehydrogenases (L-LDH) are a group of cytoplasmic holo- or hetero-tetrameric enzymes made up of four LDH subunits. Different combinations of these subunits are observed according to the tissue where they are expressed (*e.g*. heart, kidneys, lungs etc.) so that five L-LDH isozyme forms exist [5]. By contrast, d-lactate is known to be handled only by a sole mitochondrial D-LDH (EC: 1.1.1.28) encoded by the *LDHD* gene [3,4,6]. A loss of function of the D-LDH has been identified to cause severe neurological disorders, cerebellar ataxia, encephalopathy and hyperuricemia [[7], [8], [9]]. Lactic acid is furthermore deeply involved in pathophysiology of numerous conditions such as severe hypoxemia, cancer, advanced heart failure or sepsis [10] and hyperlactatemia is highly correlated with poor clinical outcomes [11]. Consequently, lactate is a mandatory biomarker in the management of septic shock, as emphasized by the Survival Sepsis Campaign [12]. Moreover, it has been strongly associated with mortality in patients with sepsis admitted in intensive care units (ICUs) [13]. Sepsis is mostly due to bacterial infections [14] and the occurrence of bacteraemia during sepsis state has been well-described so far, ranging from 30 to 50% [15]. As d-lactate is widely produced by bacteria and very fewer by human cells, we hypothesize that d-lactate could be a simple, rapid and specific biomarker of bacteraemia. To our knowledge, blood d-lactate has been measured and found elevated in patients with several gastrointestinal conditions such as short bowel syndrome, acute mesenteric ischaemia or gastrointestinal dysfunction [16,17]. In the field of infectious diseases, d-lactate was also occasionally assessed as an early indicator of infection in different matrices, such as cerebrospinal fluid, pleural fluid, ascitic fluid or synovial fluid [18]. In particular, it was identified as a promising biomarker in a context of bacterial synovitis or periprosthetic joint infections, displaying high sensitivity and specificity [[19], [20], [21], [22], [23]], as recently reviewed through a meta-analysis by Li et al. [24]. Furthermore, d-lactate was identified as a prognosis marker of 28-day mortality in ICU patients admitted in a context of septic shock [25]. Beyond this literature on osteoarticular infections, available data on d-lactate as a biomarker of infection are scarce. A possible cause of this limited material is the complexity to develop a specific analysis compatible with routine practice. As we managed to set up an automated 24/7 d-lactate assay, we have had the possibility to develop this applied research. For the first time, in this study, we aimed to assess analytical performances of d-lactate, d-lactate/l-lactate ratio and d-lactate/total lactate ratio as potential predictive biomarkers of bacteraemia.

## 2 Materials & methods

### 2.1 Study approval

This study was approved by the Ethics Committee of Rennes University Hospital Centre - Notice n°21-145.

### 2.2 Proof of concept

To evaluate the d-lactate production ability of bacteria frequently involved in bacteraemia, we used reference strains of *Escherichia coli* (ATCC 25922), *Klebsiella pneumoniae* (ATCC 700603) and *Staphylococus aureus* (ATCC 25923). For each pathogen, we inoculated three aerobic and anaerobic BCs bottles (BACTEC, Beckton Dickinson, USA) containing 5.5 mmol.L^−1^ of glucose and <0.1 mmol.L^−1^ of lactate (D and L) with 10^3^, 10^5^ and 10^7^ colony forming units (CFU).mL^−1^ suspended in 0.85% NaCl API® medium (bioMérieux, Marcy-l’Etoile, France). After a 24-h incubation, we collected the bottle content, centrifuged it twice and saved the supernatant. We then performed analysis of glucose, l-lactate and d-lactate levels for all samples.

### 2.3 Sample gathering

We performed a single-center retrospective study at Rennes University Hospital, Rennes, Brittany, France. Firstly, we screened iteratively our Laboratory Information System (LIS) for all patients who had bacteraemia (*i.*e. confirmed positive BCs) with a concomitant routine chemistry sample during a two-month period, between June and August of 2021. Blood samples for chemistry analysis were collected by peripheral venipuncture on BD vacutainer® lithium heparin blood collection tubes with separating gel (Beckton-Dickinson, Franklin Lakes, NJ, USA). The time and storage conditions were the same for the control and positive groups. NADH interference was considered negligible as collection tubes were centrifuged within an hour after venipuncture and there is no more glycolysis afterwards. The latter samples are systematically kept for seven days at +4 °C in the event that add-ons analyses are required. Thus, we were able to gather chemistry specimen leftovers belonging to the initial sampling sequence of patients for whom BCs became positive within five days. These samples represented the positive arm as the blood collected was able to trigger BCs analyser detection due to the CO_2_ production from bacteria. On the other hand, to constitute the negative (control) arm, we collected samples from the outpatient care centre of our hospital, where healthy outpatients can complete routine check-up. Overall, we gathered *n* = 131 samples from patients with confirmed bacteraemia and *n* = 97 samples from control outpatients. In the positive group, every bacterial identification performed by the bacteriology laboratory was reported as well as the delay of positivity indicated by the BD BACTEC™ system (BACTEC, Beckton Dickinson, USA).

### 2.4 L and d-lactate measurement

Plasma specimens were assessed for l-lactate using a Roche cobas® 8000 analyser with a colorimetric enzymatic method using l-lactate oxidase and a chromogenic compound for the detection. d-lactate assessment not being proposed as an automated analysis by chemistry-suppliers, we developed and validated an open canal chemistry analysis on cobas® 8000, using Roche cobas c pack MULTI (catalog reference: 05353025190; 05353025214). Briefly, this enzymatic method is based on a D-LDH reaction and a spectrophotometric detection of NADH at 340 nm, using a commercial kit from BioSenTec (Portet-sur-Garonne, France). The turnaround time on board is 10 min, technical details are available on the manufacturer's datasheet at www.biosentec.fr/docs/Tech/079-Kit-D-lactate-21031.pdf. The following criteria were assessed: limit of detection (LoD), limit of quantification (LoQ), superior limit of linearity, within and between-run imprecision. Interference of hemolysis, icterus, lipemia, l-lactate and Lactate dehydrogenase (LDH) were tested, the validation criteria for the absence of interference was a ±10% maximal deviation.

### 2.5 Statistical analysis

To assess analytical performances of d-lactate level, l-lactate level, d-lactate/total lactate and D/l-lactate ratios in predicting the occurrence of bacteraemia, Sensitivity, Specificity, Positive Predictive Value (PPV), Negative Predictive Value (NPV) and likelihood ratio were calculated. For each parameter, we established Area Under Receiver Operating Characteristic Curve (AUROC) with the Wilson-Brown method, allowing us to set optimal cut-offs. We also realized a contingency table with i) True Positive (TP), patients with confirmed bacteraemia with a test result above the cut-off; ii) False Negative (FN), patients with confirmed bacteraemia with a test result under the cut-off; iii) True Negative (TN), patients from the negative group with a test result under the cut-off; and iv) False Positive (FP), patients from the negative group with a test result above the cut-off. As d-lactate levels were supposed to be normally distributed, we used a Student *t*-test to compare d-lactate levels between the different groups. BCs time-to-positivity was also compared using *t*-test in the positive group as well. P values < 0.05 were considered statistically significant. All statistical analysis were performed with GraphPad Prism v.9.2 software (GraphPad Software, San Diego, CA, USA).

## 3 Results

### 3.1 d-lactate method characteristics

Limit of detection was 0.02 μmol.L^−1^, limit of quantification was 30 μmol.L^−1^ and superior limit of linearity was 15 mmol.L^−1^. Within-run imprecision was 0.87%, 1.24% and 1.98% for low, medium and high quality control. Between-run imprecision was 2.21%. There was no significant interference from l-lactate at the maximal concentration we tested, 15 mmol.L^−1^. Hemolysis did not interfere until 300 mg dL^−1^ of free plasmatic haemoglobin, icterus until 103 μmol.L^−1^ of unconjugated bilirubin and lipemia until a L index of 47 as determined by HIL test on Roche Cobas®, corresponding to a visually highly opalescent sample. l-Lactate Dehydrogenase did not interfere until 1000 IU.L^−1^.

### 3.2 d-lactate production by pathogens commonly involved in bacteraemia

*E. coli*, *K. pneumoniae* and *S. aureus* reference strains were screened for d-lactic acid production after an overnight incubation in blood culture medium. For all strains, at each inoculum concentration (*i.e*. 10^3^, 10^5^ and 10^7^ CFU mL^−1^), there was no residual glucose after incubation as it was all consumed during the anaerobic glycolysis. For *E. coli*, mean d-lactate level was 2 149 μmol.L^−1^ (range: 2 106–2 216), mean l-lactate level was 360 μmol.L^−1^ (range: 350–370). For *K. pneumoniae* strains, mean d-lactate level was 3 452 μmol.L^−1^ (range: 3 189–3 625) and mean l-lactate level was 457 μmol.L^−1^. For *E.coli and K. pneumonia*e, d-lactate/total lactate ratio was around 87% (85.6 ± 0.5% and 88.3 ± 0.8% respectively). For *S.aureus*, mean d-lactate level was 6 149 μmol.L^−1^ (range: 4 513–7 803), with a mean l-lactate level of 10 137 μmol.L^−1^ and a d-lactate/total lactate ratio of about 40% (37.4 ± 7.2%).

### 3.3 Retrospective analysis

**Fig. 1:**
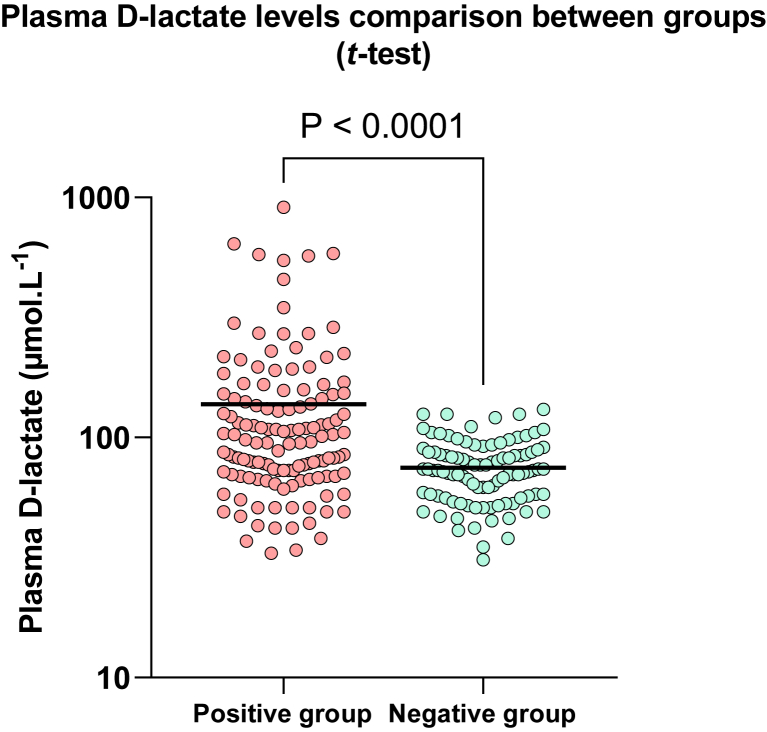
Scatter-plot of plasma d-lactate levels from the positive (red dots) and negative (green dots) groups. Mean d-lactate levels are represented by the black bold lines. Difference between d-lactate levels in both groups was evaluated by an unpaired *t*-test. (For interpretation of the references to color in this figure legend, the reader is referred to the Web version of this article.)

**Fig. 2:**
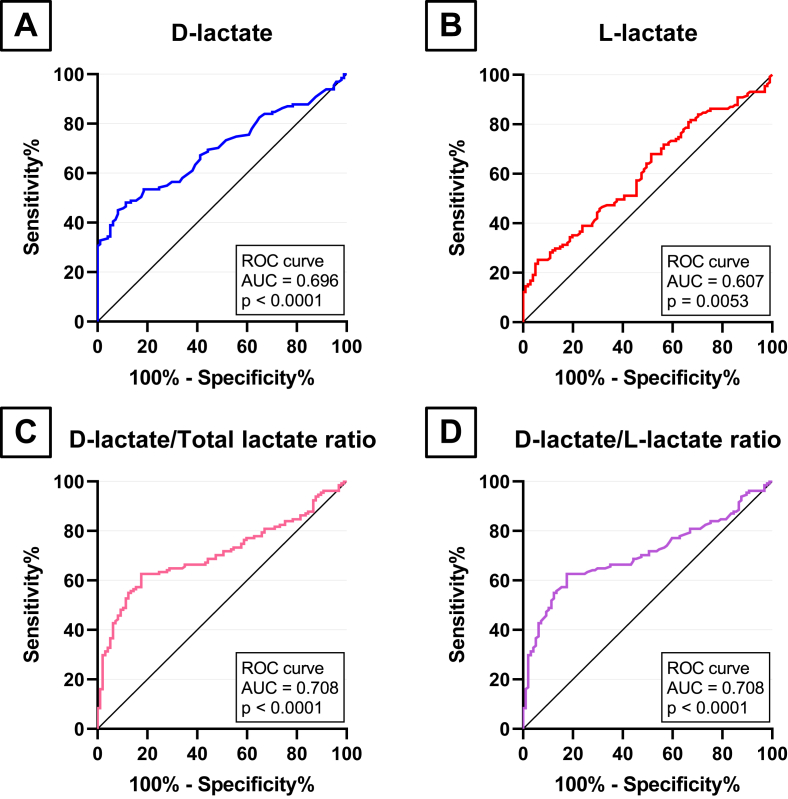
Receiver Operating Characteristic curves of **A)** Plasma d-lactate level, **B)** Plasma l-lactate level, **C)** Plasma d-lactate/Total lactate ratio and **D)** Plasma d-lactate/l-lactate ratio. AUC: Area Under Curve.

Overall, 228 specimens were collected retrospectively, 131 from patients who had bacteraemia and 97 from healthy outpatients. Bacteria considered as a contaminant we not included. Positive blood cultures were considered as such if they met the three following criteria: i) only one bottle of the set was positive; ii) a prolonged time to positivity (>20 h, reflecting a low inoculum); iii) and an identified pathogen classically considered as contaminant, such as coagulase-negative staphylococci. Samples were frozen at −80 °C until a serial analysis of l-lactate and d-lactate was performed as detailed above. Among patients with bacteraemia, 83/131 (63%) infections were due to Gram-negative bacteria, mostly *E. coli* (n = 52) and *Klebsiella* spp. (n = 13), while 48/131 (37%) were caused by Gram-positive bacteria, mainly *S. aureus* (n = 24) and *Streptococcus* spp. (n = 19). The detailed list of pathogens involved in bacteraemia is available as Supplemental Table 1. As shown in Fig. 1, d-lactate levels ranged from 33 to 909 μmol.L^−1^ in the positive group (mean = 138 μmol.L^−1^) and were statistically significantly higher than in the control group (ranging from 31 to 131 μmol.L^−1^, mean = 76 μmol.L^−1^; *P*-value for *t-*test <0.0001). l-lactate levels ranged from 1 910 to 16 770 μmol.L^−1^ in the positive group (mean = 4 857 μmol.L^−1^) and from 1 910 to 7 760 μmol.L^−1^ in the control group (mean = 3 946 μmol.L^−1^). The most specific cut-off for d-lactate level as possible was set over 131 μmol.L^−1^ based on the control group results so there was no false positive. For l-lactate, the most specific cut-off was set over 7 800 μmol.L^−1^. Cut-off for d-lactate/total lactate and D/l-lactate ratios were set to 0.0268 and 0.0275, respectively. AUROC are shown in Fig. 2 and analytical performances of each parameter are summarized in Table 1. Briefly, d-lactate/total lactate and D/l-lactate ratios both displayed a slightly wider area than d-lactate level alone (0.708 *vs* 0.696). Though, d-lactate had a 100% specificity and PPV and was therefore considered more interesting as a rule-in biomarker. Gram staining type was not found to influence d-lactate levels (*t*-test of Gram-negative *vs* Gram-positive, p = 0.55). Overall mean ± standard deviation time-to-positivity of blood cultures was 12.2 ± 6.3 h. In the FN group, it was 11.8 ± 4.1 h, which did not differ from the TP group (13.8 ± 9.9 h, p = 0.11).

**Table 1:** Predictive value of biomarkers at admission for the development of bacteraemia.

Biomarker	AUROC (95% CI)	Cut-off	Sensitivity%	Specificity%	Likelihood Ratio	PPV%	NPV%	P value
d-lactate (μmol.L^−1^)	0.696 (0.629–0.763)	131 μmol.L^−1^	**31** (23.3–38.9)	**100** (96.2–100)	/	**100** (91.2–100)	**52** (44.5–58.6)	<0.0001
l-lactate (μmol.L^−1^)	0.607 (0.535–0.679)	7800 μmol.L^−1^	**12** (8.7–18.9)	**100** (96.3–100)	/	**100** (80.6–100)	**46** (40.2–53.4)	0.0053
d-lactate/total lactate ratio	0.708 (0.640–0.775)	0.0268	**30** (22.6–38.1)	**98** (92.8–99.1)	14.4	**95** (83.9–99.1)	**51** (43.7–57.9)	<0.0001
d-lactate/l-lactate ratio	0.708 (0.641–0.775)	0.0275	**30** (22.6–38.1)	**98** (92.8–99.6)	14.4	**95** (83.9–99.1)	**51** (43.7–57.9)	<0.0001

AUROC: Area Under Reciever Operating Curve; PPV: Positive predictive value; NPV: Negative predictive value.

## 4 Discussion

Bacterial infections are the first cause of sepsis and a priority in primary care [14]. Bloodstream infections may be transient or persistent undetected until jeopardizing patient's condition. It is well addressed that the earliness and the appropriateness of treatment is highly related with survival [26]. Thus, it would be of major interest to have simple highly specific biomarkers to quicken the initiation of antibiotics. The gold standard in characterizing bacteraemia relies on BCs analysis. The process is based on the seeding and incubation of enriched medium with blood to promote an exponential growth that allows detection and thereafter identification of causative pathogens. BCs is a highly-sensitive and highly-specific method to determine the aetiology of a bloodstream infection and therefore represents the reference diagnosis method to do so [[27], [28], [29]]. However, BCs in daily practice usually requires a time of positivity that commonly exceed 10 h, sometimes even more. Recent technical advances allow to perform identification directly on positive BCs bottles without needing to subculture the medium on agar plate with as good analytical performances as the standard practice [30,31]. As a result, turnaround times are drastically reduced to a ‘within a day period’ with a rapid identification protocol [31]. Still, this kind of protocol is not available in routine practice and so there is a loss of chance between daytime and night-time positive samples. Moreover, several pre-analytical specifications such as blood sampling volume are required to perform efficient blood cultures, without which diagnosis accuracy can be considerably impacted [[32], [33], [34]]. Once the positivity is confirmed, several innovating methods are raising to improve identification or antibiotic susceptibility testing [28,33]. Nevertheless, these phenotypic methods operate after the confirmation of blood culture positivity and therefore depend on the time to detection. Here, we propose a rapid, highly specific biomarker to fasten the detection of bacteraemia and so potential sepsis, with a 24/7 rendering within an hour from venepuncture. Considered as a metabolic waste for decades, lactate is now well described to have pleiotropic roles such as in energy regulation, cellular growth, inflammation, immuno-modulation or wound healing [35], but the role of d-lactate remains uncertain due to its low levels in human body. In 2002, a mitochondrial D-LDH (EC: 1.1.1.28) encoded by *LDHD* gene was first characterized [3,4,6], suggesting a physiological specific way of detoxifying d-lactate involving the methylglyoxal pathway [36]. This mechanism was previously thought to be carried out by D-2-hydroxy acid dehydrogenase. Recent studies highlighted that d-lactate dehydrogenase is mandatory in human metabolism, although d-lactate is present to a very small extent in healthy individuals whereas it can be massively produced by bacteria, fungi or even protozoan parasites [[7], [8], [9],^37^,^38^]. Hence, d-lactate could represent a biomarker of interest but it is an underexplored metabolite [2], mostly because it may be difficult to characterize with standard chemistry analyses. Several descriptions of d-lactate methods can be found in literature, often developed for gastrointestinal indications [39,40]. These methods can be fully automated, however, technical pitfalls must be evaluated. For example, elevated l-lactate and/or l-lactate dehydrogenase (LDH) levels may lead to falsely elevated d-lactate levels by generating non-specific NADH production [41,42]. These interferences must be tested by laboratories willing to validate d-lactate kits and addressed if needed. To prove our concept, we needed to demonstrate that pathogens commonly involved in bacteraemia produced a D-LDH and therefore produced d-lactate. Based on the previous work by Garvie and coworkers [43], we selected strains frequently found in bacteraemia to perform an *in vitro* inoculation of BCs bottles. After a 24-h incubation in an enriched medium, *E. coli* and *K. pneumoniae* strains almost fully metabolized glucose into d-lactate (d-lactate/l-lactate ratio of 87%) whereas *S. aureus* displayed an equilibrated lactate repartition (d-lactate/l-lactate ratio of 40%). This might suggest that lactate racemase (*i.e*. the bacterial enzyme catalysing the interconversion of L- and d-lactate) equilibrium could be in favour of d-lactate in Gram-negative bacteria in comparison to Gram-positive. However, we did not find a significant difference of d-lactate levels between these two groups (p = 0.55). The d-lactate level cut-off chose to reach a maximal specificity is 131 μmol.L^−1^, approximatively four-fold the limit of quantification of the method, which was determined as the concentration allowing a 10% imprecision: 40 μmol.L^−1^. This setting led to only a slightly decreased sensitivity. By contrast, for d-lactate/total lactate and D/l-lactate ratios, setting the cut-off to obtain 100% of specificity was not relevant regarding the significant loss of sensitivity. As expected, l-lactate analytical performances were not as efficient as those of d-lactate, displaying a very low sensitivity (12%) when the cut-off is over 7800 μmol.L^−1^ to reach maximal specificity. This last result strengthens our hypothesis of a bacterial specificity of d-lactate given that there are plenty causes of lactate acidosis [44]. In this study, we observed a heterogeneity in the positive group with a wide range of d-lactate levels (33–909 μmol.L^−1^) while dispersion was tighter in the negative group (31–131 μmol.L^−1^). It would be of interest to explore whether inoculum can be correlated to d-lactate production. The strains isolated from positive BCs in our study were consistent with epidemiology of bacterial pathogens responsible for bloodstream infections*.*

Finally, the timing for starting empirical antibiotic therapy is challenging, mostly based on severity. The use of d-lactate in clinical practice could confirm an early detection of bacteraemia and allow a quick initiation of antibiotics in patients suspected from infection, who do not strictly meet all criteria of the Sepsis-3 definition [12]. In addition, it has been shown that the time of administering effective antibiotics is correlated with prognosis in sepsis [45]. Consequently, a rapid measurement of d-lactate, combined with its high specificity, could represent a relevant and helpful tool in order to initiate antibiotic therapy more quickly and appropriately.

## 5 Study limitations

This study has limitations. First, this single-center retrospective study is strictly based on biological data as it was designed to assess analytical performances of d-lactate and ratios to predict bacteraemia. Thus, further validation of these biomarkers from a clinical standpoint will be necessary in order to enable the use of d-lactate in clinical routine. For instance, it would have been convenient to be aware of conditions that may cause false positive such as diabetes decompensation or gut dysbiosis. Besides, L- and d-lactate assessment were performed on frozen samples that were gathered between one and three days after venopuncture. We did not quantify and therefore cannot guarantee the absence of D-LDH activity at 4 °C even though it is thought to be marginal. Finally, the production of d-lactate has only been evaluated in a small number of bacteria, which does not allow to extrapolate on a wide range of genus or species and we did not included samples from non-bacteraemic septic patients, which would have been interesting.

## 6 Conclusion

This work is the first to our knowledge to emphasize d-lactate as a highly-specific early detection biomarker of bacteraemia. Further prospective studies are needed to confirm whether it could find a place in bloodstream infection characterization by warning clinician prior to the blood culture positivity and rule-in septic patients. Kinetics studies or biomarker combination research resulting in diagnosis score could help to raise the low sensitivity of d-lactate in blood that represents a weakness at the moment.

## Author contribution statement

Charles René LEFEVRE: Conceived and designed the experiments; Performed the experiments; Analyzed and interpreted the data; Contributed reagents, materials, analysis tools or data; Wrote the paper.

Adrien Turban: Performed the experiments; Analyzed and interpreted the data; Wrote the paper.

Malo Penven: Bénédicte Langlois: Maxime Pawlowski: Nicolas Collet: Caroline Piau: Analyzed and interpreted the data; Wrote the paper.

Céline René: Performed the experiments; Contributed reagents, materials, analysis tools or data.

Vincent Cattoir: Claude Bendavid: David Luque Paz: Conceived and designed the experiments; Analyzed and interpreted the data; Wrote the paper.

## Data availability statement

Data will be made available on request.

## Declaration of competing interest

As mentioned in the manuscript, the authors declare they have no competing financial or personal interest that could have appeared to influence the work reported in this paper.
