# Supplementary material for: Early detection of plasma d-lactate: Toward a new highly-specific biomarker of bacteraemia?
Source: Heliyon. 2023 May 21;9(6):e16466. doi: 10.1016/j.heliyon.2023.e16466 (PMC10230201; doi:10.1016/j.heliyon.2023.e16466)
Supplement: Multimedia component 1 [file mmc1.docx]

| **Culture identification** | **Number of isolates** |
| --- | --- |
| Gram positive cocci | **45** |
| *Staphylococcus aureus* | 24 |
| *Streptococcus dysgalactiae* | 6 |
| *Streptococcus anginosus group* | 4 |
| *Enterococcus faecalis* | 3 |
| *Streptococcus mitis-oralis group* | 3 |
| *Streptococcus agalactiae* | 2 |
| *Enterococcus faecium* | 1 |
| *Streptococcus gallolyticus* | 1 |
| *Streptococcus intermedius* | 1 |
| *Streptococcus pneumoniae* | 1 |
| *Streptococcus pyogenes* | 1 |
| Gram negative bacilli | **82** |
| *Escherichia coli* | 52 |
| *Klebsiella pneumoniae* | 9 |
| *Enterobacter cloacae* | 4 |
| *Pseudomonas aeruginosa* | 3 |
| *Klebsiella oxytoca* | 3 |
| *Morganella morganii* | 2 |
| *Klebsiella aerogenes* | 1 |
| *Parabacteroides sp.* | 2 |
| *Bacteroides vulgatus* | 1 |
| *Delftia acidovorans* | 1 |
| *Proteus vulgaris* | 1 |
| *Raoultella ornithinolytica* | 1 |
| *Salmonella sp.* | 1 |
| *Serratia marcescens* | 1 |
| Gram positive bacilli | **4** |
| *Clostridium ramosum* | 1 |
| *Lactobacillus delbrueckii* | 1 |
| *Listeria monocytogenes* | 1 |
| *Arcanobacterium sp.* | 1 |
| Total | **131** |

**Table S1.** List of pathogens involved in bacteraemia.
